# Cross-sectional study assessing sleep behavior and musculoskeletal symptoms in elite junior badminton athletes

**DOI:** 10.1097/MD.0000000000038698

**Published:** 2024-07-05

**Authors:** Kaja Skare, Bjarke Viberg, Per Hölmich, Stewart Kerr, Niels Christian Kaldau

**Affiliations:** a Department of Orthopaedic Surgery and Traumatology, Odense University Hospital, Odense, Denmark; b Department of Orthopedic Surgery, Sports Orthopedic Research Center – Copenhagen, Copenhagen University Hospital, Amager & Hvidovre Hospital, Copenhagen, Denmark; c Department of Clinical Research, University of Southern Denmark, Odense, Denmark; d Life Fit Wellness, Healthcare & Exercise Centre, Falkirk, Scotland, United Kingdom.

**Keywords:** adolescent, athletes, badminton, musculoskeletal pains, sleep

## Abstract

Sleep inadequacy has previously been associated with increased risk of injury and reduced performance. It is unclear if sleep disorders are associated with musculoskeletal symptoms, which may be a predictor of serious injury and affect performance. The aim was therefore to assess sleep behavior in elite junior badminton players and its association to musculoskeletal symptoms. In 2018, players at the World Junior Badminton Championship completed the Athlete Sleep Behavior Questionnaire and a modified version of the World Olympic Association Musculoskeletal Health Questionnaire. Participants were categorized with poor or moderate/good sleep behavior as the independent variable. Musculoskeletal symptoms were the primary outcome and was categorized using yes/no questions. Relevant musculoskeletal symptoms were defined as pain higher than 30 mm Numeric Rating Scale pain score or more than 30 minutes of joint stiffness a day. Group comparison was performed using chi-square analysis and logistic regression for primary outcome adjusted for age, sex, ethnicity, previous injury, training load, and resting days. Of the 153 participants, 28% reported poor sleep scores. There was no difference between poor and moderate/good sleep score concerning demographic variables such as sex, age, ethnicity, previous injury, training load, and resting days. There were 27% with current musculoskeletal symptoms but with no difference in groups between poor and moderate/good sleep score (*P* = .376). This yielded an adjusted odds ratio of 1.23 (95% confidence intervals 0.52; 2.90). Twenty-eight percent of the participants reported poor sleep behavior. Twenty-seven percent experienced current musculoskeletal symptoms. We found no statistical differences in reported musculoskeletal symptoms when comparing athletes with poor sleep behavior to athletes with moderate/good sleep behavior.

## 1. Introduction

### 1.1. Background

Sleep is a vital aspect of overall health and well-being^[1]^ and adequate sleep may play a key role for athletes due to the physical demands of their sport.^[2]^ Junior elite athletes face unique challenges in terms of balancing training, competition, education, and other responsibilities. As elite athletes they are often required to train, compete, and travel at unfavorable times and sometimes across various time-zones, all of which may impact their sleep quality and quantity.^[3]^ Hence, sleep disturbances may play a role in the high injury burden amongst junior elite athletes,^[4]^ where nearly 50% of elite junior badminton players sustain a serious injury at one point in their career.^[5]^

Insufficient sleep may lead to physical- and cognitive impairments,^[6]^ which may impact injury rates and performance.^[2,3]^ A study from Brazil on elite athletes have found poor sleep quality to be associated with musculoskeletal complaints,^[7]^ potentially leading to injury or time-loss from activity. According to Milewski et al,^[8]^ sleep deprivation is one of the strongest predictors for injury, finding that junior athletes sleeping <8 hours per night were at 1.7 times higher risk of injury than their counterparts sleeping the recommended 8 hours or more. Furthermore, sleep inadequacy may reduce various aspects of athletic ability, ultimately effecting optimal performance.^[2]^

It has yet to be sufficiently assessed how sleep behavior affects musculoskeletal symptoms. To the authors knowledge there is no data investigating junior elite badminton players sleep behavior, nor the relation to musculoskeletal symptoms. If there is a relationship between sleep inadequacy and musculoskeletal symptoms, it may be easy to target sleep behavior^[9]^ and hopefully prevent injuries and improve performance.^[6]^ This article is part of the Global Health Badminton Study initiated by the Badminton World Federation (BWF) to evaluate the best junior elite badminton players in world.

### 1.2. Objectives

The aim of this study is to assess sleep behavior in elite junior badminton players and its association to musculoskeletal symptoms. A potential problem with sleep behavior should lead to future studies investigating causalities and the results in this study could serve as a reference point for future studies with elite adolescent athletes.

## 2. Methods

### 2.1. Study design

As part of a larger study on elite junior badminton athletes,^[5]^ a cross sectional study was designed using questionnaires to collect information related to sleep behavior and musculoskeletal health, including basic demographic information. Reporting was performed according to the STROBE guidelines.^[10]^ Study methods were conformed to the Helsinki declaration and approved by Danish Data Protection Agency (VD-2018-409). After evaluation by the Capitol Region Committee on Health Research Ethics in Denmark it was determined that no ethical approval was required. Due to the global nature of the study, the BWF Legal Team evaluated and approved the study design as well as the translations of the questionnaires.

### 2.2. Setting

The study took place during the Junior World Championship in badminton in Markham, Canada, November 8th to 17th 2018. The recruitment period was during the championship. Data was collected and stored using REDCap electronic data capture tools.^[11]^ The questionnaires were available electronically online in English. Paper versions were available in English, Spanish, French, Indonesian, Korean, Chinese, and Thai. Non-English questionnaires were coded, making them easily convertible to the English version in REDCap.

### 2.3. Participants

All participants competing in the championship were invited to participate in the study via national team leaders and coaches. Information about the study was distributed at team leader meetings and via email through the BWF to the forementioned. Since it was a youth championship, all those invited were under the age of 19. A written consent was obtained to meet the eligibility criteria. Those without a written consent were excluded from the study. Out of 436 athletes, 164 accepted the invitation. Asian countries received questionnaires in paper format in their native language. Participants from Spanish and French speaking countries were also offered the possibility to receive questionnaires in their native language. The remaining participants were provided a link to an online English questionnaire. In addition, it was possible for all participants to complete the questionnaire at the coauthors on-site testing station at the championship. Participants were contacted by email for follow-up and given until May 1st, 2019, to answer questions that were not completed.

### 2.4. Variables, quantitative variables, and measurements

Sleep behavior was the independent variable, defined as sleep behavior over the last month. Based on score thresholds from the questionnaire, participants were categorized with “good,” “moderate” or “poor” sleep behavior. Due to the primary outcome focusing on “poor” sleep behavior, “moderate” and “good” sleep behavior were combined as one group. Sleep hygiene questions were self-reported and based on the Athlete Sleep Behavior Questionnaire (ASBQ). The ASBQ is an 18-item survey developed to evaluate sleep behavior of athletes. Items in the questionnaire include afternoon naps, usage of stimulants when training, late-night exercise, alcohol consumption, bedtime and waking variation, nighttime thirst, nighttime soreness, usage of light emitting technology before bed time, worries of sporting performance and/or other issues when in bed, utilization of sleeping pills, sleep interruption due to bathroom visits, snoring and/or muscle twitching, sleeping environment, and subjective effect of travel on sleep-wake routine. The ASBQ is a valid measurement tool when compared to other established sleep questionnaires and has high levels of test-retest reliability.^[12]^ Responses of never, rarely, sometime, frequently and always were ranked using a 5-point Likert scale. The sum of these responses was converted to a global score ranging from 18 to 90. The ASBQ defines score thresholds for “good” (global score ≤ 36) and “bad” (global score ≥ 42) sleep behavior.^[12]^ The questionnaire is developed and validated for adult elite athletes. Due to a limited number of other studies, it has yet to be validated for the junior population. Likewise, it has not been validated in other languages than English.

Current musculoskeletal symptom(s) was the dependent variable, focusing on pain and joint stiffness:

1. Pain was defined as currently experiencing pain (for most days of the last month) in at least one joint (yes/no). Due to the subjective measure of pain, its intensity was assessed on a Numeric Rating Scale from 0 (no pain) to 10 (strongest pain).^[13]^ A study conducted on low back pain patients found that Visual Analog Scale intensity ratings above 30 mm out of 100 mm range had significant direct effects on sleep disturbance.^[14]^ The score threshold in this study was therefore set at >30 mm on the Numeric Rating Scale.
2. Joint stiffness was defined as the sensation of difficulty moving a joint or the apparent loss of range of motion of a joint. As joint stiffness is a normal condition after hard physical exercise and is common for elite athletes to experience during shorter periods, the author group decided that only those experiencing stiffness with a duration of more than 30 minutes a day were found pathological. Participants reporting duration <30 minutes were therefore included in the category with those that did not report any stiffness. Musculoskeletal health questions were self-reported and based on the World Olympic Association Musculoskeletal Health Global Questionnaire. The questionnaire was divided into 2 sections, separating serious previous injury and current musculoskeletal symptoms, with our focus on the latter. The questionnaire was modified to fit the population similarly as in previous studies within the same population.^[5]^ The modified questionnaire has not been validated in other studies.

Demographic variables were categorized based on a previous study conducted on the same population.^[5]^ Sex was categorized as “Male,” “Female” or “Unknown,” and ethnicity as “Asian” or “Non-Asian.” Age was categorized as under or over 17 years old as this was the mean age of the participants.

The questionnaire included 5 options for current anti-inflammatory drugs and/or pain relievers usage (daily, weekly, once a month, very seldom, not at all). Participants reporting utilizing anti-inflammatory drugs and/or pain relievers daily or weekly were categorized as “Yes,” while reports of usage once a month, very seldom, or not at all, were categorized as “No.” Previous significant injury was defined as an injury lasting 30 days or more and causing a reduction in training capacity.^[5]^ Those still experiencing lasting limitations and pain from the previous injury were found relevant and grouped as such. Training load categories were based on a previous study assessing prevalence of severe injury in the same population,^[5]^ where hours of badminton training and physical training per week have been combined as one group to total training hours per week for practical reasons. Resting days were defined as number of rest days per week the past 30 days and categorized as either “0–1 days” or “≥2 days.”

### 2.5. Statistical methods, sample size, and bias

This was a cross-sectional study with elite junior badminton players and a sample size calculation was not possible as the cohort’s size was predetermined. It is therefore possible that there is low power. Descriptive analysis on the study population is tabulated according to participant characteristics as number and percentage categorized by the 2 comparison groups: poor and moderate/good sleep behavior. The main results are similarly presented as number and percentages with 95% confidence intervals (CI). Comparison between groups was performed with Person chi-squared test and 95% CI.

Multiple potential confounders were identified for adjustment. A Japanese study of junior badminton players found that female gender and higher age significantly increase injury rates,^[15]^ making it reasonable to think they could similarly influence musculoskeletal symptoms and is therefore adjusted for. Ethnicity is adjusted as badminton is a popular sport in Asian countries, which may lead to a different and more elite training culture than in non-Asian countries.^[5]^ Models were also fitted for those with lasting limitation and pain from previous injury, training load, and rest days, as it is reasonable to think that these factors could influence current musculoskeletal symptoms.^[16,17]^

The fit of all logistic regression model was evaluated by Chi-Square goodness of fit test. Crude odds ratio (OR) was calculated for musculoskeletal symptoms that was used as the dependent variable. Adjusted OR was calculated adjusting for sex, age, ethnicity, lasting pain and/or limitations from previous significant injury, training load, and number of resting days.

## 3. Results

### 3.1. Participants and descriptive data

**Figure 1.**
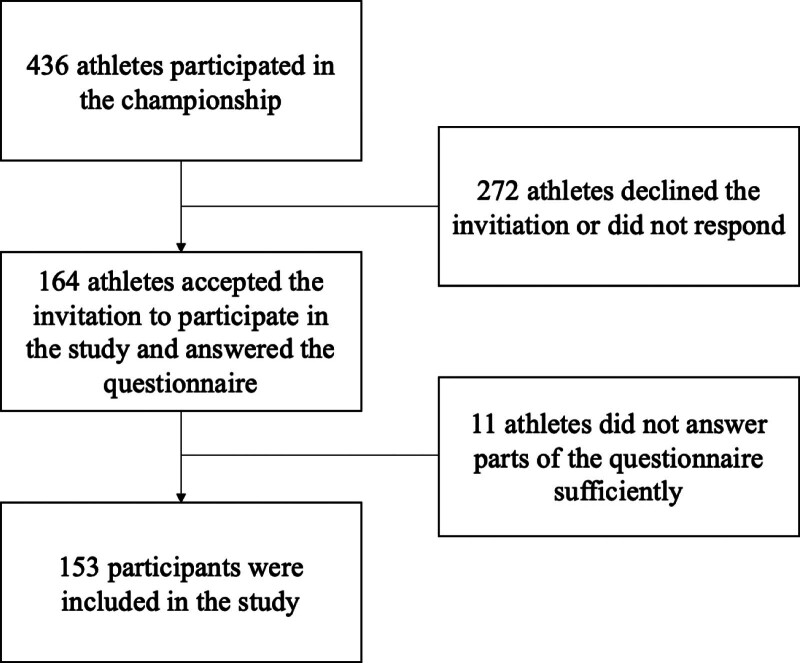
Flow diagram of participant inclusion.

There were 164 participants that answered the questionnaires, of which 11 were excluded during analysis due to lack of response in the ASBQ. The final cohort consisted of 153 participants (Fig. 1). Nearly one third (28%) of the participants were classified with poor sleep behavior (Table 1). There was a slight overweight of male participants (55%), nearly 80% were over the age of 17, and more than 60% were non-Asian. Three players reported usage of anti-inflammatory and/or pain relievers. There were no statical differences between groups concerning demographic variables, lasting pain and/or limitations from previous significant injury, pain medication usage, training load, and number of resting days (Table 1)*.*

**Table 1:** Demographic data.

	Study population	Sleep score				*P*
		*Poor*		*Moderate/good*		
	n	n	%	n	%	
**Participants**	153	43	28	110	72	
**Sex**						.102
* Male*	85	18	21	67	79	
* Female*	65	24	37	41	63	
* Unknown*	3	1	33	2	67	
**Age**						.152
* <17*	33	6	18	27	82	
* 17+*	120	37	31	83	69	
**Ethnicity**						.062
* Asian*	57	11	19	46	81	
* Non-Asian*	96	32	33	64	67	
**Anti-inflammatory drugs and/or pain relievers**						.857
* Yes*	3	1	3	2	2	
* No*	126	36	97	90	98	
**Previous significant injury**						.158
* No*	78	18	23	60	77	
* Yes*	75	25	33	50	67	
* Still experiences pain or functional limitation*	36	15	42	21	58	
**Training load**						.476
* 0–19 hours per week*	50	15	30	35	70	
* 20–39 hours per week*	68	21	31	47	69	
* 40*+ *hours per week*	35	7	20	28	80	
**Resting days**						.644
* 0–1 day*	118	32	27	86	73	
* 2*+ *days*	32	10	31	22	69	

### 3.2. Main results

There were 27% (42/153) who reported current musculoskeletal symptoms (Table 2). Of those with current musculoskeletal symptoms, 33% had poor sleep score compared to 67% with moderate/good sleep score (*P* = .376). There was no significant difference in the OR of having current musculoskeletal symptoms in participants with poor sleep scores when compared to participants with moderate/good sleep scores (OR = 1.41, 95% CI [0.66; 3.05]). Nor were there significant differences in OR after adjusting for potential confounders (OR = 1.23, 95% CI [0.52; 2.90]).

**Table 2:** Current musculoskeletal symptoms.

Sleep score		Current musculoskeletal symptoms		*P* value	Crude logistic regression *OR (95% CI*)	Adjusted** logistic regression *OR (95% CI*)
		No	Yes			
Poor*	*n*	29	14	0.376	1.41 (0.66; 3.05)	1.23 (0.52; 2.90)
	*%*	26	33			
Moderate/good	*n*	82	28			
	*%*	74	67			

* *Reference category*.

** Adjusted for age, sex, ethnicity, training load, limitation/pain of previous injury, and resting days.

## 4. Discussion

### 4.1. Interpretation

Our study suggests that a high percentage of elite junior badminton players have suboptimal sleep behavior, which is consistent with the few other studies also using the ASBQ. A study on varsity athletes of various sports in Canada found the prevalence of poor sleep behavior to be 62%.^[18]^ While this study was not specifically conducted on junior athletes, the mean ages of the participants (20 years old) is close to the mean age of 17 in our study. However, there may be differences in sleep parameters between junior and adult elite athletes,^[19]^ highlighting the need for more research on this young population. Sleep behavior may be affected by a multitude of factors, of which the causality was not feasible to assess in this study. However, the authors find it contiguous to suggest unfavorable training- and competition times, as well as travels, to be of particular importance. Sleep behavior leading up to a major competition may furthermore be altered due to anxiety and stress.^[20]^ Our findings therefore suggests that athletes, coaches, and parents should be mindful of sleep behavior and consider implementing sleep interventions^[9]^ during competitive season.

Given the desire to constantly push themselves to achieve their goals, it is not surprising our study found a high prevalence of musculoskeletal symptoms in these junior elite athletes. The finding raise concern as they were gathered near a championship when athletes may be inclined to disregard symptoms and persevere. Our results are consistent with those of other studies, such as Malmborg et al,^[21]^ who reported daily pain in one-third of junior athletes attending a sports school. Owoeye et al^[22]^ recently found preseason pain in one-fourth of apparently healthy collegiate soccer and basketball players. These findings suggest that athletes may be willing to compete despite experiencing pain, which is in line with a study on German elite junior athletes.^[23]^ It is important to note that while pain is a common occurrence in sports, the mentality of “no pain, no gain” may be harmful. Prolonged pain may be a sign of underlying tissue damage and may increase the risk of more serious injuries that can have both short- and long-term effects on performance, as well as overall health.^[24]^ Pain management and an awareness of early signs of injury may therefore be important to learn for the young athlete to be able to train continuously without getting injured.

A recent narrative review reported high levels of noninflammatory and/or pain reliever usage among junior elite athletes.^[25]^ Surprisingly, our study found very low levels of usage in this population. This may be due to a more professional and healthy management of the young athletes in the badminton community. Another possible explanation for these contradicting findings is a general growing interest in doping prevention, particularly evident over the past 5 years, in both junior and senior athletes.^[26]^ Due to the low usage, we did not find it relevant to include this variable in the statistical analysis.

Both sleep behavior and musculoskeletal symptoms in the junior elite population is highly understudied, hence, comparable data is scarce. One study in Brazil found elite athletes (mean age = 21) with poor sleep quality defined by the Pittsburg Sleep Quality Index, to have more frequent musculoskeletal complaints.^[7]^ This is not in concordance with our findings. A possible explanation for this discrepancy is that sleep behavior may not always reflect sleep quality. Another possible explanation is the limited number of participants in our study, as about 4 times as many participants would be required to show a 10 percentage-points difference. Furthermore, the reciprocal relationship between poor sleep and musculoskeletal symptoms raises an etiological question that is challenging to study and answer. Some research has found that multiple sleep parameters are associated with reduced pain tolerance in nonathlete adults.^[27]^ On the contrary, it has been suggested that sleep problems in adult elite athletes may be caused by pain during sleep.^[7]^ The conflicting results, and/or the lack thereof, emphasize the need for further research on the relationship between sleep and musculoskeletal complaints to better understand the underlying mechanisms. Our study provides insights into this area in junior elite athletes, complementing the limited research currently available.

### 4.2. Limitations

We acknowledge there are limitations to consider when interpreting the results of our study:

i Players with previous serious injuries and ongoing musculoskeletal problems may not have qualified for the championship. These players could have contributed to a higher study population; however, we do would not expect an increase in statistical difference.
ii Self-reported measures of musculoskeletal symptoms are subject to interindividual variability.^[28]^ Additionally, athletes tend to overestimate their sleep duration when using subjective measures.^[29]^
iii Variable thresholds and sleep behavior categories were based on expert recommendations and are likely to reflect real-world patterns of sleep behavior. We have carefully considered the potential impact of these choices on our data and believe that any effects are minimal.
iv The musculoskeletal health questionnaire has not been validated, and the ASBQ has not been validated for juniors. Neither have been validated after translation into native languages. Furthermore, the questionnaires do not include sleep duration, making it difficult to compare with other sleep studies in the athletic population.

### 4.3. Key results

In conclusion, we found a high prevalence of poor sleep behavior suggesting elite junior badminton players may be at risk for sleep inadequacy and should be mindful of sleep behavior during competitive season. We also found that pain and/or joint stiffness is frequent in elite junior badminton players, and it may be important for the young athlete to learn about pain management. However, we found no significant difference in musculoskeletal symptoms when comparing athletes with poor sleep behavior with athletes with good/moderate sleep behavior. The impact of poor sleep behavior and musculoskeletal symptoms on well-being and performance has not been investigated in this study and may be interesting to study in the future.

## Acknowledgments

Mark King contributed to the study design and methodology. The authors would like to thank the players for participation in the study.

## Author contributions

**Conceptualization:** Niels Christian Kaldau, Stewart Kerr.

**Data curation:** Bjarke Viberg.

**Formal analysis:** Bjarke Viberg.

**Funding acquisition:** Niels Christian Kaldau.

**Methodology:** Niels Christian Kaldau, Per Hölmich, Stewart Kerr.

**Project administration:** Niels Christian Kaldau, Stewart Kerr.

**Supervision:** Per Hölmich.

**Writing – original draft:** Kaja Skare.

**Writing – review & editing:** Niels Christian Kaldau, Bjarke Viberg.
